# Risk factors for tuberculosis smear non-conversion in Eden district, Western Cape, South Africa, 2007–2013: a retrospective cohort study

**DOI:** 10.1186/s12879-016-1712-y

**Published:** 2016-08-02

**Authors:** Mandla Mlotshwa, Natasha Abraham, Moira Beery, Seymour Williams, Sandra Smit, Margot Uys, Carl Reddy, Andrew Medina-Marino

**Affiliations:** 1 South African Field Epidemiology Training Programme, National Institute for Communicable Diseases, Johannesburg, South Africa; 2 Epidemiology Research Unit, Foundation for Professional Development, Pretoria, South Africa; 3 School of Health System and Public Health, University of Pretoria, Pretoria, South Africa; 4 Department of Health, George, Western Cape South Africa

**Keywords:** Tuberculosis, Smear non-conversion, Risk factors, Treatment outcomes, Trends, South Africa

## Abstract

**Background:**

Tuberculosis (TB) continues to be a major global health problem. While progress has been made to improve TB cure rates, South Africa’s 76 % smear-positive pulmonary TB (PTB) case cure rate remains below the WHO target of 85 %. We report on the trends of TB smear non-conversion and their predictors at the end of an intensive phase of treatment, and how this impacted on treatment outcomes of smear-positive PTB cases in Eden District, Western Cape Province, South Africa.

**Methods:**

Routinely collected, retrospective data of smear-positive PTB cases from the electronic TB register in Eden District between 2007 and 2013 was extracted. Non-conversion was defined as persistent sputum smear-positive PTB cases at the end of the two or three month intensive phase of treatment. Chi-square test for linear trend and simple linear regression analysis were used to analyse the change in percentages and slope of TB smear non-conversion rates over time. Risk factors for TB non-conversion, and their impact on treatment outcomes, were evaluated using logistic regression models.

**Results:**

Of 12,742 total smear-positive PTB cases included in our study, 12.8 % (*n* = 1627) did not sputum smear convert; 13.3 % (1411 of 10,574) of new cases and 9.9 % (216 of 2168) of re-treatment cases. Although not statistically significant in either new or re-treatment cases, between 2007 and 2013, smear non-conversion decreased from 16.4 to 12.7 % (slope = −0.60; 95 % CI: −1.49 to 0.29; *p* = 0.142) in new cases, and from 11.3 to 10.8 % in re-treatment cases (slope = −0.29; 95 % CI: −1.06 to 0.48; *p* = 0.376). Male gender, HIV co-infection and a >2+ acid fast bacilli (AFB) smear grading at the start of TB treatment were independent risk factors for non-conversion (*p* < 0.001). Age was a risk factor for non-conversion in new cases, but not for re-treatment cases. Non-conversion was also associated with unsuccessful treatment outcomes (*p* < 0.01), including treatment default and treatment failure.

**Conclusions:**

Smear-positive PTB cases, especially men and those with identified risk factors for non-conversion, should be closely monitored throughout their treatment period. The South African TB control program should invest in patient adherence counselling and education to mitigate TB non-conversion risk factors, and to improve conversion and TB cure rates.

**Electronic supplementary material:**

The online version of this article (doi:10.1186/s12879-016-1712-y) contains supplementary material, which is available to authorized users.

## Background

Tuberculosis (TB) remains a major public health problem worldwide, with an estimated 9 million new cases and 1.5 million deaths reported in 2013 [1]. The latest estimates by the World Health Organization (WHO) TB Global Report indicates that the Asia and African regions accounted for more than two-third of new cases in 2013, with the highest absolute number of reported incident TB cases in India, China, Nigeria, Pakistan, Indonesia and South Africa [2]. Globally, South Africa ranks third for TB burden of disease [1]. In 2010, the incidence of TB in South Africa was 981 per 100,000 population, but there was a gradual decline to 860 per 100,000 in 2013 due to the increased rollout of the national anti-retroviral therapy (ART) program [2–5]. In 2013, there were 410,000–520,000 cases of TB reported in South Africa, with more than 250,000 of these cases co-infected with human immunodeficiency virus (HIV) [2]. While HIV has fuelled the incidence of TB in South Africa, the emergence of drug resistant TB is threatening to destabilize the already constrained South African National TB Control Program (NTCP) [6, 7].

Although considerable progress has been made in the past decade to improve TB cure rates and treatment outcomes, the South African TB cure rate of 76 % remains below the WHO target of 85 % [1, 3, 8]. Furthermore, South Africa was among the highest TB burden countries unable to achieve the highly ambitious millennium development goals (MDG) of halving their TB prevalence and mortality compared to the 1990 baseline, and reducing the incidence of TB cases to 1 case per 1,000,000 population per year by 2015 [2]. This highlights the need to continue strengthening the existing TB control programs to improve TB case finding, treatment outcomes and mitigate the spread of TB infection.

In South Africa, the NTCP routinely collects TB data in an electronic TB register (ETR.Net). ETR.Net functions as a surveillance tool to estimate the burden of TB, guide planning, evaluate local and national level TB control programs and inform policy decisions on resource allocation [9]. In line with WHO recommendations, the NTCP evaluates sputum smear conversion at the end of an intensive phase of treatment, 2 months for new cases and 3 months for re-treatment cases [9]: smear conversion is a strong predictor of treatment outcomes [10, 11]. Surveillance of smear conversion rates is used as an operational indicator for clinical management of smear-positive pulmonary TB (PTB) cases, effectiveness of TB treatment and the performance of the directly observed treatment short-course (DOTs) strategy [9]. In South Africa, the average national rate of smear conversion increased from 45 % in 2004 to 67 % in 2013, with the TB cure rate for smear-positive PTB cases rising from 62 % in 2006 to 76 % in 2012 [3, 8].

In 2013, among the South Africa’s nine provinces, KwaZulu Natal had the highest TB incidence (864 per 100,000 population), followed by Eastern Cape (792 per 100,000 population) and Western Cape (711 per 100,000 population) [8]. Of Western Cape’s six districts, Eden is the second largest and has a TB incidence rate of 806 per 100,000 population [8]. Furthermore, Eden is the one of ten key districts piloting the roll-out of the South African National Health Insurance (NHI) program [8].

In support of South Africa’s efforts to achieve the global target of 85 % TB cure rate, we undertook a retrospective analysis of smear-positive PTB cases over a seven-year period in Eden district. We sought to investigate the trends and risk factors for smear non-conversion at the end of an intensive phase of treatment and their impact on treatment outcomes.

## Methods

### Study design

**Fig. 1:**
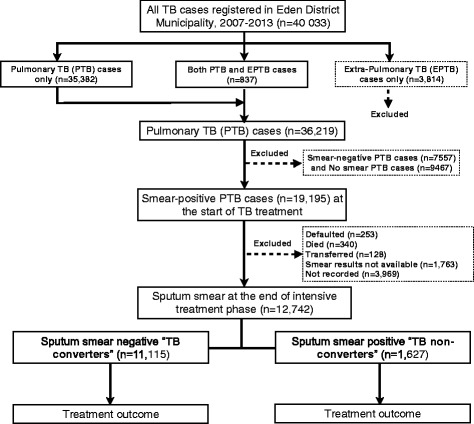
Study flow chart showing the selection of pulmonary tuberculosis cases (*n* = 12,742) in the electronic TB register Eden District, Western Cape Province, 2007–2013

A retrospective cohort of study of PTB cases was done using routine TB programmatic data in the ETR.Net in Eden District, Western Cape Province, South Africa, between 2007 and 2013. Only smear-positive PTB cases, with or without extra-pulmonary TB (EPTB), with documented smear results at the end of 2- or 3-month of an intensive phase of treatment (Fig. 1) were included. Exclusion criteria were: (i) extra-pulmonary TB cases only, (ii) smear-negative PTB cases, (iii) PTB cases with no smear results at the start of treatment, and (iv) smear-positive PTB cases with no smear results at the end of 2- or 3-month intensive phase of treatment.

### Study setting

All cases of TB registered in the ETR.Net database in Eden District between 2007 and 2013 were evaluated for inclusion. Eden has an estimated population of 585,833 people and a population density of 25.1 persons per km^2^ [3]. With an estimated TB incidence of 806 per 100,000 population in 2013, Eden District is above the incidence rate of 711 per 100,000 population in Western Cape Province and 593 per 100,000 population in South Africa [8]. Eden district is divided into seven sub-districts: Bitou, George, Hessequa, Knysna, Kannaland, Mossel Bay and Oudtshoorn. Eden’s TB control program is based on a network of its sub-districts primary and community health clinics (PHC and CHC’s), that provide TB testing, care and treatment. Treatment guidelines include (i) a standardized 6-month rifampicin based regimen for new cases and an 8-month regimen for re-treatment cases, and (ii) the evaluation of acid fast bacilli (AFB) sputum smear conversion from positive to negative at the end of the intensive phase of treatment: 2 months for new cases and 3 months for re-treatment cases.

### Definitions

As per South African NTCP Practical Guidelines [9], TB cases were defined as follows: New TB case: an individual with a smear-positive AFB result who has never had treatment for TB or taken anti-TB treatment drugs for less than 4 weeks. Re-treatment TB case: an individual with a smear-positive AFB result who has taken TB treatment for 4 weeks or more in the past and either relapsed, defaulted or had treatment failure.

TB converters: PTB patient with smear-positive AFB result prior to treatment initiation, whose AFB smear result is negative at the end of an intensive phase of treatment (2-month for new cases and 3-month for re-treatment cases).

TB non-converters: PTB patient with smear-positive AFB result prior to treatment initiation whose AFB smear result remains positive at the end of an intensive phase of treatment (2-month for new cases and 3-month for re-treatment cases).

Treatment outcomes were defined according to the South African NTCP Practical Guidelines [9] and the 2013 WHO definitions and reporting framework for TB [12].

Successful treatment outcome: PTB cases who completed treatment and who were declared cured. Cured: PTB cases with completed treatment and smear-negative AFB result at 1 month prior to the completion of treatment and on at least one previous occasion. Completed: PTB cases with completed treatment and no AFB smear result at 1 month prior to the completion of treatment and on at least one previous occasion.

Unsuccessful treatment outcome: PTB cases who died, defaulted treatment, failed treatment, or were not evaluated. Failed: PTB cases with a smear-positive AFB result prior to treatment initiation and who remained smear-positive at five months or later during treatment. Died: PTB cases who died for any reason during the course of treatment. Defaulted: PTB cases whose treatment was interrupted for two or more consecutive months. Not evaluated: PTB cases for whom no treatment was assigned, whose treatment outcome was unknown, or that transferred to a clinic outside of Eden District.

### Data collection

Extracted ETR.Net database variables included age, sex, HIV status, disease classification and category, sub-districts and clinic facilities, treatment regimen and outcomes, and sputum smear results. Data were exported into a Microsoft Excel spreadsheet and analysed using STATA 13.0 (Stata Corporation, College Station, Texas, USA).

### Statistical analysis

PTB cases were categorized into TB converters and non-converters based on sputum smear results status at the end of an intensive phase of treatment. Descriptive statistics were used to compare baseline characteristics of TB converters and non-converters. Continuous variables were tested for normality using Shapiro-Wilk’s W test and expressed as mean ± standard deviation (SD) or median and interquartile (IQR) ranges depending on the distribution of data. Categorical variables were expressed as frequencies (n) and percentages (%), and analysed by the Pearson’s Chi-squared (*χ*^2^) test or two-sided Fischer’s exact test. Chi-square test for linear trend was used to analyse the percent change in TB non-converters over time. Simple linear regression analysis was used to assess the percent change in the slope of TB non-converters over time.

Univariate and multivariate logistic regressions were used to identify predictors or factors independently associated with TB smear non-conversion at the end of 2- or 3-month phase of intensive TB treatment, and assess the effect of TB smear non-conversion on treatment outcomes. A manual forward stepwise selection of the variables with a threshold of *p* < 0.25 in the univariate analysis were incorporated into a multivariate logistic regression model to adjust for potential confounders such as age and gender. In a logistic regression analysis, data were expressed as unadjusted and adjusted odd ratios (aOR) and 95 % confidence interval (CI). All statistical tests were two-sided and a *p* ≤ 0.05 was considered statistically significant.

### Ethical review

Ethical clearance and approval for this study was provided by the University of Pretoria, Faculty of Health Sciences Research Ethics Committee (Ethic Reference no: 236/2015) and the Western Cape Provincial Department of Health (Reference no: WC_2015RP27_678). TB patient privacy and confidentiality were strictly maintained through removing personal identifying information and allocating unique patient identifiers.

## Results

### Description of the study cohort

During 2007–2013, a total of 40,033 TB cases were registered in the Eden District ETR.Net database: 35,382 (88 %) PTB cases only, 837 (2 %) PTB and EPTB cases and 3814 (10 %) EPTB cases only (Fig. 1). EPTB only cases were excluded from our analysis. Of the 36,219 PTB cases, 19,195 (53 %) were smear-positive at the start of TB treatment. Of these, only 12,742 (66 %) had documented smear results at both treatment initiation and at the end of the intensive phase of treatment (group 1); 6453 (34 %) did not have documented smear results at the end of the intensive phase of treatment (group 2). Comparisons of baseline characteristics between the two groups of PTB cases revealed a significance differences for age, gender, HIV status, and TB classification (*p* < 0.0001, Table 1). No significant differences were observed between the two groups of PTB cases in terms of disease classification (*p* = 0.061), pre-treatment smear grading (*p* = 0.053) and sub-districts (*p* = 0.887). Of the 12,742 PTB cases included in the final analysis: mean age was 35 ± 13.1 years, 7612 (60 %) were male and 2720 (21 %) were HIV positive (Table 1). Of the 12,742 PTB cases included in our study, 10,574 (83 %) were classified as new cases of TB and 2168 (17 %) were re-treatment cases.

**Table 1:** Baseline characteristics of sputum smear-positive pulmonary tuberculosis (PTB) cases in Eden District, Western Cape Province, 2007–2013

Variables	All PTB cases		PTB cases with smear results^a^		PTB cases without smear results^a^		
	n	%	n	%	n	%	*p* value
Age, years, mean (SD)	36 ± 12.8	-	35 ± 13.1	-	37 ± 12.3		*p* < 0.0001
Gender							*p* < 0.0001
Female	7556	39.35	5130	40.26	2424	37.56	
Male	11,641	60.65	7612	59.74	4029	62.44	
HIV status							*p* < 0.0001
Negative	13,086	68.17	9047	71.00	4039	62.59	
Positive	4256	22.17	2720	21.35	1536	23.80	
Unknown	1853	9.65	975	7.65	878	13.61	
Disease classification							*p* = 0.061
PTB	19,024	99.11	12,640	99.20	6384	98.93	
PTB and EPTB	171	0.89	102	0.80	69	1.07	
TB classification							*p* < 0.0001
New	12,833	66.86	10,574	82.99	2259	35.01	
Re-treatment	6362	33.14	2168	17.01	4194	64.99	
Smear conversion status^a^							
Negative	11,115	87.23	11,115	87.23	-	-	
Positive	1627	12.77	1627	12.77	-	-	
Pre-treatment smear grading							*p* = 0.053
Scanty	1670	8.70	1101	8.64	569	8.82	
AFB+	3991	20.79	2665	20.92	1326	20.55	
AFB++	3983	20.75	2659	20.87	1324	20.52	
AFB+++	8575	44.67	5712	44.83	2863	44.37	
Not recorded	976	5.08	605	4.75	371	5.75	
Sub-district							*p* = 0.887
Bitou	1662	8.66	1119	8.78	543	8.41	
George	7101	36.99	4731	37.13	2370	36.73	
Hessequa	1245	6.49	818	6.42	427	6.62	
Kannaland	940	4.90	615	4.83	325	5.04	
Knysna	2088	10.88	1388	10.89	700	10.85	
Mossel Bay	3090	16.10	2028	15.92	1062	16.46	
Oudtshoorn	3069	15,99	2043	16.03	1026	15.90	

*SD* Standard deviation, *TB* tuberculosis, *AFB* Acid fast bacilli, *PTB* Pulmonary TB, *EPTB* Extra-pulmonary TB

^a^sputum smear results at the end of 2- or 3- month intensive phase of treatment

### Trends in TB sputum smear non-conversion

**Fig. 2:**
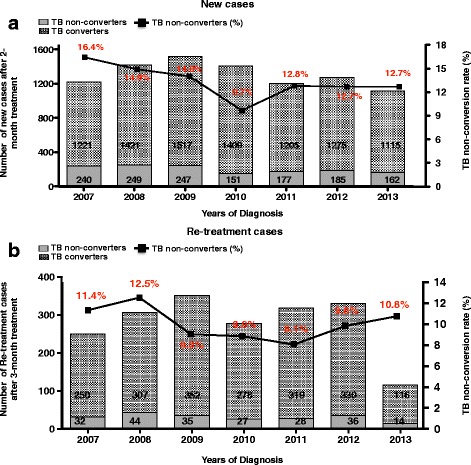
Trends in tuberculosis sputum smear non-conversion rate in Eden District, Western Cape Province, 2007–2013. New (**a**) and Re-treatment (**b**) cases TB non-converters at the end of 2- and 3-month intensive phase of treatment

From 2007 to 2013, a total 1627 (12.8 %) PTB cases were classified as TB non-converters: 13 % (1411 of 10,574) of new cases and 9.9 % (216 of 2168) of re-treatment cases were non-converters. The annual percent change of TB non-conversion in new cases significantly decreased from 16.4 % in 2007 to 12.7 % in 2013 (chi-square trend: 13.66, *p* < 0.001, Fig. 2a). In re-treatment cases, the annual percent change of TB non-conversion decreased from 11.4 % in 2007 to 10.8 % in 2013 (Fig. 2b), however, this decrease was not statistical significance (chi-square trend: 1.49, *p* = 0.222). The percentage change in the slope of TB non-conversion over time in a simple linear regression revealed a non-significant decline in new cases of TB (slope = −0.60, 95 % CI: −1.49 to 0.29, *p* = 0.142, Table 2). A similar trend was observed in the slope of TB non-conversion in re-treatment cases (slope = −0.29, 95 % CI: −1.06 to 0.48, *p* = 0.376).

**Table 2:** Percentage changes in the slope of TB non-conversion in Eden district, Western Cape Province, 2007-2013

Sub-districts	New TB cases			Re-treatment TB cases		
	Slope	95 % CI	*p* value	Slope	95 % CI	*p* value
Bitou	0.17	−0.81 to 1.15	0.6717	0.61	−2.16 to 3.39	0.5941
George	−0.45	−1.87 to 0.97	0.4507	−0.55	−1.72 to 0.62	0.2796
Hessequa	−0.83	−1.85 to 0.21	0.0946	−0.25	−4.86 to 4.37	0.8943
Kannaland	−1.68	−3.29 to −0.07	0.0430	1.94	−2.22 to 6.09	0.285
Knysna	−0.70	−2.01 to 0.61	0.2261	0.19	−1.76 to 2.16	0.8069
Mossel Bay	−0.73	−2.14 to 0.67	0.2393	−0.85	−1.96 to 0.26	0.106
Oudtshoorn	−0.69	−0.81 to 1.15	0.6717	−0.36	−3.16 to 2.40	0.7470
Eden District	−0.60	−1.49 to 0.29	0.1421	−0.29	−1.06 to 0.48	0.3766

Of new TB cases, a statistically significant annual decrease in TB non-conversion was observed in Kannaland sub-district (chi-square trend: 5.07, *p* = 0.0243): no significant decrease was observed in Eden’s six other sub-districts (see Additional file 1: Figure S1). Linear regression further revealed a statistically significant decline in the slope of TB non-conversion in Kannaland sub-district (Table 2, slope = −1.68, 95 % CI: −3.29 to −0.07, *p* = 0.0430). In re-treatment cases, neither the decreases in non-conversion in George, Hessequa and Mossel Bay sub-districts, nor the increases in Knysna, Kannaland and Oudtshoorn sub-districts were significant (see Additional file 1: Figure S1). In Bitou sub-district, neither the decrease in non-conversion from 2007 to 2012, nor the increase in 2013 was statistically significant. Analysis of the changes in slope of TB non-conversion in re-treatment cases were not significant in any of Eden’s seven sub-districts (Table 2).

### Predictors of TB smear non-conversion

In a univariate analysis, factors found to be independently associated with TB non-conversion in new cases were (i) age categories > 15 years, (ii) being male, (iii) HIV infection, (iv) smear grading (> AFB+) prior to treatment, and (v) being treated in George and Mossel Bay sub-districts (Table 3). In contrast, new cases of TB that received treatment between 2008 and 2013 were at a lower risk for non-conversion at the end of 2-month intensive phase of treatment than those treated in 2007 (*p* < 0.01, Table 3). After adjusting for potential confounders in a multivariate logistic regression analysis, age categories > 15 years, being male, HIV infection and smear grading (> AFB+) remained independently associated with non-conversion at the end of 2-month intensive phase of treatment (Table 3). Among re-treatment cases, risk factors for non-conversion at the end of the 3-month intensive phase of treatment were (i) being male, (ii) HIV infection and (iii) smear grading (>AFB+) prior to treatment (Table 4). The risk of non-conversion at the end of intensive phase of treatment was 66 % in male and 53 % in HIV-TB co-infected patients. In addition, re-treatment cases with smear grades of AFB++ and AFB+++ prior to treatment were at 3 and 5 times more likely not to smear convert at the end of the intensive phase of treatment, respectively (Table 4). After adjusting for potential confounders, being male, HIV infection and smear grades of AFB++ and AFB+++ remained independently associated with smear non-conversion at the end of the intensive phase of treatment (Table 4).

**Table 3:** Predictors of tuberculosis smear non-conversion in new tuberculosis cases

Variable	Total	Non-converters		Univariate analysis			Multivariate analysis		
	N	n	%	Crude OR	95 % CI	*p*-value	Adjusted OR	95 % CI	*p*-value
Age, years	10,506	1411							
0–14	157	5	3.18	Reference					
15–30	4325	419	9.69	3.26	1.33–7.99	0.010	2.32	0.94–5.73	0.067
31–45	3801	596	15.68	5.65	2.31–13.8	<0.000	3.77	1.53–9.31	0.004
46–60	1816	325	17.89	6.63	2.69–16.27	<0.000	4.65	1.88–11.5	0.001
>60	407	66	16.22	5.88	2.32–14.89	<0.000	4.56	1.79–11.6	0.001
Gender	10,574	1411							
Female	4380	451	10.29	Reference			Reference		
Male	6194	960	15.49	1.59	1.42–1.80	<0.000	1.44	1.27–1.63	<0.000
HIV	10,574	1411							
Negative	7538	979	12.98	Reference			Reference		
Positive	2192	341	15.56	1.23	1.08–1.41	0.002	1.41	1.22–1.62	<0.000
Unknown	844	91	10.78	0.81	0.64–1.02	0.069	0.74	0.58–0.94	0.013
Pretreatment smear grading	10,574	1411							
Scanty	936	47	5.02	Reference			Reference		
AFB+	2271	173	7.76	1.56	1.12–2.17	0.009	1.64	1.17–2.28	0.004
AFB++	2227	240	10.78	2.28	1.65–3.15	<0.000	2.42	1.75–3.35	<0.000
AFB+++	4613	904	19.59	4.61	3.41–6.24	<0.000	4.83	3.56–6.55	<0.000
Not recorded	527	47	8.92	1.85	1.22–2.82	0.004	1.86	1.22–2.84	0.004
Year									
2007	1461	240	16.43	Reference			Reference		
2008	1670	249	14.91	0.89	0.73–1.08	0.244	0.81	0.66–0.99	0.044
2009	1764	247	14.00	0.83	0.68–1.00	0.056	0.75	0.62–0.92	0.006
2010	1560	151	9.68	0.54	0.44–0.67	0.000	0.52	0.41–0.64	0.000
2011	1382	177	12.61	0.75	0.61–0.92	0.007	0.71	0.57–0.88	0.002
2012	1460	185	12.67	0.74	0.60–0.91	0.004	0.68	0.55–084	0.000
2013	1277	162	12.69	0.74	0.59–0.92	0.006	0.75	0.60–0.94	0.012
Sub-district	10,574	1411							
Bitou	913	103	11.28	Reference			Reference		
George	3919	533	13.60	1.24	0.98–1.54	0.062	1.20	0.95–1.51	0.119
Hessequa	6.83	93	13.62	1.24	0.92–1.67	0.160	1.28	0.94–1.74	0.117
Kannaland	504	68	13.49	1.23	0.88–1.70	0.222	1.18	0.84–1.66	0.312
Knysna	1131	143	12.64	1.14	0.87–1.49	0.347	1.13	0.85–1.48	0.398
Mossel Bay	1699	241	14.18	1.29	1.01–1.66	0.037	1.28	1.00–1.66	0.050
Oudtshoorn	1725	230	13.33	1.21	0.94–1.54	0.132	1.18	0.92–1.53	0.192

**Table 4:** Predictors of tuberculosis smear non-conversion in re-treatment tuberculosis cases

Variable	Total	Non-converters		Univariate analysis			Multivariate analysis		
	N	n	%	Crude OR	95 % CI	*p*-value	Adjusted OR	95 % CI	*p*-value
Gender	2168	216							
Female	750	54	7.2	Reference			Reference		
Male	1418	162	11.42	1.66	1.21–2.29	0.002	1.68	1.21–2.33	0.002
HIV	2168	216							
Negative	1509	133	8.81	Reference			Reference		
Positive	528	68	12.88	1.53	1.12–2.08	0.007	1.81	1.32–2.51	0.000
Unknown	131	15	11.45	1.34	0.76–2.36	0.314	1.38	0.77–2.46	0.268
Pretreatment smear grading	2168	216							
Scanty	165	5	3	Reference			Reference		
AFB+	394	17	4.31	1.44	0.52–3.97	0.479	1.56	0.56–4.32	0.389
AFB++	432	38	8.79	3.08	1.19–7.98	0.020	3.54	1.36–9.20	0.009
AFB+++	1099	152	13.83	5.14	2.07–12.7	<0.000	5.79	2.33–14.4	0.002
Not recorded	78	4	5.13	1.73	0.45–6.63	0.424	1.99*	0.52–7.68	0.315

### Effects of TB smear non-conversion and treatment outcome

Of new cases, the overall treatment success rate was significantly lower in non-converters (78.7 %) compared to converters (85.7 %; *p* < 0.0001; see Additional file 2: Figure S2 and Table 5); unsuccessful treatment outcome was significantly higher in non-converters (21.3 %) compared to converters (14.4 %, *p* < 0.0001; see Additional file 2: Figure S2 and Table 5). A similar trend was also observed in re-treatment cases when treatment success and unsuccessful treatment outcomes were compared between converters and non-converters (see Additional file 2: Figure S2 and Table 6).

**Table 5:** Effect of TB non-conversion on treatment outcome in new tuberculosis cases

Variable	Non-converters		Converters		Univariate analysis			Multivariate analysis		
	n	%	n	%	Crude OR	95 % CI	*p*-value	Adjusted OR	95 % CI	*p*-value
Cured	939	66.55	6809	74.31	Reference			Reference		
Completed	172	12.19	1040	11.35	1.19	1.00–1.42	0.042	1.18	0.99–1.42	0.060
Died	27	1.91	129	1.41	1.51	0.99–2.31	0.052	1.33	0.86–2.05	0.205
Failed	54	3.83	76	0.83	5.15	3.61–7.35	<0.000	4.46	3.07–6.49	<0.000
Defaulted	91	6.45	380	4.15	1.73	1.36–2.20	<0.000	1.82	1.42–2.33	<0.000
Not evaluated	128	9.07	729	7.96	1.27	1.04–1.55	0.018	1.32	1.07–1.62	0.009

**Table 6:** Effect of TB non-conversion on treatment outcome in re-treatment tuberculosis cases

Variable	Non-converters		Converters		Univariate analysis			Multivariate analysis		
	n	%	n	%	Crude OR	95 % CI	*p*-value	Adjusted OR	95 % CI	*p*-value
Cured	112	51.85	1358	69.57	Reference			Reference		
Completed	35	16.20	254	13.01	1.67	1.12–2.49	0.012	1.60	1.06–2.41	0.023
Died	8	3.70	53	2.72	1.83	0.85–3.94	0.123	1.73	0.78–3.80	0.174
Failed	19	8.80	28	1.43	8.23	4.45–15.19	0.000	8.14	4.25–15.57	<0.000
Defaulted	22	10.19	126	6.45	2.12	1.29–3.46	0.003	1.96	1.18–3.25	0.009
Not evaluated	20	9.26	133	6.81	1.82	1.09–3.03	0.020	1.86	1.11–3.13	0.019

In a univariate analysis, failure of new cases to smear convert at 2-month was significantly associated with “failed” (OR: 5.15, 95 % CI: 3.61–7.35, *p* < 0.0001), “defaulted” (OR: 1.73, 95 % CI: 1.36–2.20, *p* < 0.0001) and “not evaluated” (OR: 1.27, 95 % CI: 1.04–1.55, *p* < 0.0001) treatment outcomes (Table 5). After adjusting for age, gender, HIV and pre-treatment smear grading, this association remained significant (Table 5). After adjusting for gender, HIV status and pre-treatment smear grading among re-treatment TB cases, non-conversion at 3-month was significantly associated with “completed” (aOR: 1.60, 95 % CI: 1.06–2.41, *p* = 0.023), “failed” (aOR: 8.14, 95 % CI: 4.25–15.57, *p* < 0.0001), “defaulted” (aOR: 1.96, 95 % CI: 1.18–3.25, *p* = 0.009) and “not evaluated” (aOR: 1.86, 95 % CI: 1.11–3.13, *p* = 0.019) treatment outcomes (Table 6).

## Discussion

In the present study, we report on the trends and predictors of sputum smear non-conversion at the end of 2- or 3-month intensive phase of treatment and their impact on treatment outcomes. Our analysis of 12,742 PTB cases with documented smear results at the end of the intensive phase of treatment, between 2007 and 2013, showed that the overall sputum smear non-conversion rates of new and re-treatment cases were 13.3 and 9.9 % respectively. This translates into smear conversion rates of 86.7 % in new cases and 90.1 % in re-treatment cases, which is significantly higher than the South African national average of 66.7 % and NTCP’s target of 85 % for the performance of TB control program at provincial and district levels [3, 8, 9]. However, it is worth noting that this data only represent one-third (35 %) of PTB cases that were recorded in ETR.Net database. Consequently, we cannot rule out that missing data in the ETR.net database may have impacted the calculated smear conversion rate in Eden District. This said, our findings are comparable to prior studies conducted in Cameroon [13] and Burkina Faso [14], and significantly lower than those reported in Morocco [15], India [16] and Ghana [17]. The reasons for this high smear conversion rate observed in Eden District compared to the South African national average rate of 66.7 % are unclear, but could be partly explained by (i) increased funding for TB control program [18, 19], (ii) improved access and quality of care [20, 21] and (iii) massive anti-retroviral therapy (ART) roll-out programme in the Western Cape Province [4, 5]. We note that there are fewer re-treatment cases in 2013 than in other years. While the exact reason for this is unclear, incomplete data reporting and low follow-up rate of smear results in re-treatment cases may be factors. Additional research is needed to understand this result and how loss to follow of smear results can be improved.

Eden District ETR.Net data reveal a non-significant decline in the trend of smear non-conversion rates in new and re-treatment cases of TB during the period of 2007 to 2013. This data extend and confirms the finding of Heunis et al. [22] in Free State Province, South Africa, who demonstrated a gradual decline in gender related trends of 2-month smear non-conversion in new smear-positive PTB cases from 2003 to 2009. Parallel to this finding is the report by Massyn et al. [3], demonstrating a steady increase in trends of smear conversion rates of PTB cases in South Africa from 44 % in 2004 to 68.1 % in 2010. Furthermore, recent data from Nanoo et al. [5] also showed that the annual incidence of microbiologically confirmed PTB cases (per 100,000 population) in South Africa have declined from 848 in 2008 to 774 in 2012. Of considerable interest in relation to the findings of Nanoo et al. [5] is the observation that Western Cape Province was the first province to show a decline in incidence of confirmed PTB cases, with a 4–9 % year-on-year decrease from 2007 until 2012. A host of TB control programme activities, interventions and policies that were implemented over the period of 2007–2013 in the Western Cape Province could possibly account for the observed decline in trends of smear-positive PTB cases non-conversion rates in Eden District [6, 20, 23–25]. These include improved case management of TB, multifaceted TB screening programs, introduction of community based tracing teams, better coordination of DOTs, integration of HIV/TB services at districts and sub-district levels and rollout of anti-retroviral therapy (ART) [6, 20, 23–25]. However, it was not possible to ascribe the decline of smear non-conversion rate in Eden District to any particular activity or intervention. Whether this decline in trends of TB non-conversion in Eden District is generalizable to other districts in the Western Cape Province warrants further investigation.

Smear non-conversion at the end of 2- or 3-month intensive phase of treatment is widely considered a poor predictor of treatment outcome [11, 26]. Thus, understanding factors that affect smear non-conversion is essential to improve TB cure rate and the success of TB control program. Previous studies have found that age, male gender, smoking, alcohol abuse, diabetes, high pre-treatment smear grading, HIV status, lung cavitation, anaemia, previous history of TB treatment, poor quality of anti-TB drugs, suboptimal dosage of anti-TB drugs and presence of TB drug resistant strain to be significant factors affecting TB smear non-conversion at the end of intensive phase of treatment [15, 27–29]. In our study, we identified age, high pre-treatment smear grading, male gender and HIV infection to be independently and significantly associated with smear non-conversion at the end of intensive phase treatment. Our findings are similar to those reported by Banu Rekha et al. [30] and Caetano Mota et al. [31], which showed an association of age, male and higher pre-treatment grading with the lack of smear conversion at the end of intensive phase of treatment. This is further corroborated by the retrospective cohort study conducted in Free State Province, South Africa, which showed that age, pre-treatment smear grading and TB disease classification were significantly associated with failure to smear convert at the end of intensive phase of treatment [32]. More recently, Djouma et al. [33] showed that high pre-treatment smear grading and years of treatment (2009 to 2012) were independently associated with delayed smear conversion in a retrospective cohort study in Cameroon. In our study, we also observed years of treatment (2008 to 2013) to be significantly associated with risk of smear non-conversion at end of 2-month intensive phase of treatment. We attribute this to the substantial improvement in the TB control programme in South Africa ranging from implementation of household-based case finding [34], introduction of DOTs [35] and community-based tracing team [23], intensified case finding [36], provision of ART in HIV-TB co-infected patients [5], and scaling up of Xpert MTB/RIF for early diagnosis of TB [37, 38].

Our findings also demonstrate a dose response effect of pre-treatment smear grading on smear non-conversion at the end of intensive phase of treatment. Pulmonary TB cases with baseline smear of AFB +++ in new and re-treatment cases of TB were 5 and 6 times more likely not to smear convert at end of intensive phase of treatment. High bacillary load at the start of treatment could possibly reflect the presence of lung cavitation and thus severity of disease, which has been shown to be associated with TB treatment failure and relapse, and the development of TB drug resistance [39]. Moreover, there is a direct association between the presence of lung cavitation in TB patients and high bacillary load in their sputum [39, 40]. The lack of smear conversion at the end of an intensive phase treatment with male gender has been associated with alcohol consumption and smoking habits [41]. However, in our study we could not establish this association as this data is not routinely collected in the South African electronic TB register. Nevertheless, our findings are consistent with several studies which have demonstrated a significant association between male gender and failure to smear convert at the end of the intensive phase of treatment [15, 30, 42].

In addition to male gender, HIV infection was also found to be significantly associated with failure to smear convert at the end of intensive phase of treatment. This finding is in agreement with the study of Kayigamba et al. [43], but in contrast with studies that have reported that HIV negative TB patients were more likely to not smear convert at the end of intensive phase of treatment compared to HIV positive TB patients [32] or those whose HIV status is not known [44]. The association of being HIV negative in the latter study was attributed to specialist-driven treatment adherence training given to HIV positive patients [32]. Other studies have also found no association between HIV and lack of smear conversion at the end of an intensive phase of treatment [13, 30, 31]. Taken together, these data raise a question on the role of HIV status as a predictor of smear non-conversion. Given these conflicting findings, additional studies are needed to address the impact of HIV status on TB smear conversion rates.

Finally, our results show that smear non-conversion at the end of the 2- or 3-month intensive phase of treatment was independently associated with the programmatically defined outcomes of “treatment defaulter”, “failure” and “not evaluated”. Similar findings have been seen in studies from Cameroon [13, 27, 33], India [45] and Nigeria [46]. These findings and our results highlight a need for more aggressive and cost-effective strategies to reduce smear non-conversion rates at the end of intensive phase of treatment and improve TB treatment outcomes. Our findings also highlight the need for better surveillance and data collection on TB indicators.

Our study has several limitations. Specifically, the use of retrospective routinely collected data in the electronic TB register excludes the collection of other potential risk factors. We also excluded data for which patients had smear negative results (20.9 %), no documented smear results at the start of the treatment (26.1 %) and end of intensive phase of treatment (17.8 %). Furthermore, we could not evaluate the impact of DOTs on smear conversion rate and treatment outcomes as this data was poorly captured in the electronic TB register database. Finally, as South Africa maintains a separate database for drug resistant TB cases, we could not evaluate the impact of drug resistance on smear non-conversion rates in this study.

## Conclusions

Our main findings documents (i) a non-significant decline in smear non-conversion trends of new and re-treatment cases in Eden District during 2007–2013, (ii) and that smear-positive PTB cases who are male, older than 30 years, HIV positive and have had high pre-treatment smear grading were more likely not to smear convert at the end of intensive phase of treatment. We also showed that during the study period from 2008 to 2013, years of treatment was significantly associated with lower risk of smear non-conversion in new cases. Lastly, failure to smear convert at the end of an intensive phase of treatment was significantly associated with unsuccessful treatment outcome in new and re-treatment cases.

Our findings highlight an urgent need in the district TB control program to explore strategies that will mitigate the risk of non-conversion and inform implementation of interventions to maximize indicators for TB treatment success rate. Targeted interventions should focus on (i) strengthening TB treatment adherence counselling and support, and (ii) improve follow-up of high-risk groups, especially older men living in the communities with the highest burden of TB and HIV. TB adherence clubs, support groups and peer counselling for those at risk for non-conversion may maximize patient adherence. Such programs may be adapted from previously implemented TB and/or HIV adherence programs [47–51]. Investing in mobile technology systems that fast-track laboratory test results and that reminds patients to take their TB medication would assist the TB control activities in the district to improve follow-up and TB indicators for treatment outcomes. Well-designed prospective studies are needed to formulate robust interventions. Furthermore, implementation research will inform strategies that should be incorporated into TB control programs to maximize TB treatment outcomes.

## Abbreviations

AFB, acid fast bacilli; aOR, adjusted odd ratio; ART, anti-retroviral therapy; CHC’s, community health clinics; DOTs, directly observed treatment on short course; EPTB, extra-pulmonary TB; ETR.Net, electronic TB register; HIV, human immunodeficiency virus; IQR, interquartile range; MDG, millennium development goals; MTB/RIF, mycobacterium tuberculosis rifampicin resistance; NHI, national health insurance; NTCP, South African national TB control program; OR, odd ratio; PTB, pulmonary TB; SD, standard deviation; TB, tuberculosis; WHO, World Health Organisation

## Additional files

Additional file 1: Figure S1. Trends in tuberculosis sputum smear non-conversion rate in Eden sub-districts, Western Cape Province, 2007–2013. Chi-square trend for changes in the percentage of TB non-conversion and their respective *p* values are shown above in the graphs. (PDF 114 kb) Additional file 2: Figure S2. Comparisons of treatment outcome in tuberculosis non-converters and converters of new and re-treatment TB cases. (PDF 49 kb)
