# Supplementary figures and images for: Risk factors for tuberculosis smear non-conversion in Eden district, Western Cape, South Africa, 2007–2013: a retrospective cohort study
Source: BMC Infect Dis. 2016 Aug 2;16:365. doi: 10.1186/s12879-016-1712-y (PMC4971671; doi:10.1186/s12879-016-1712-y)

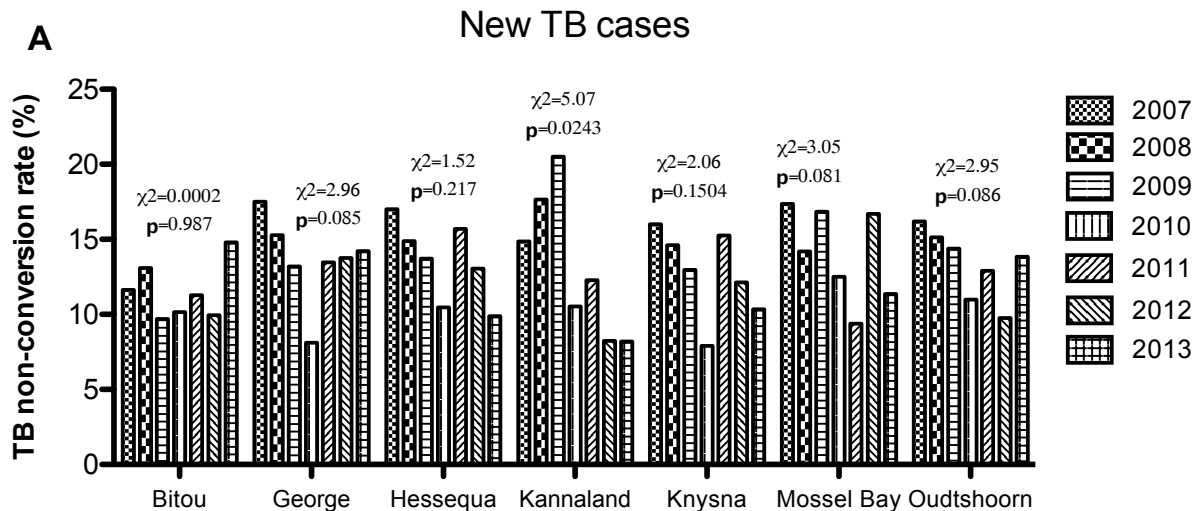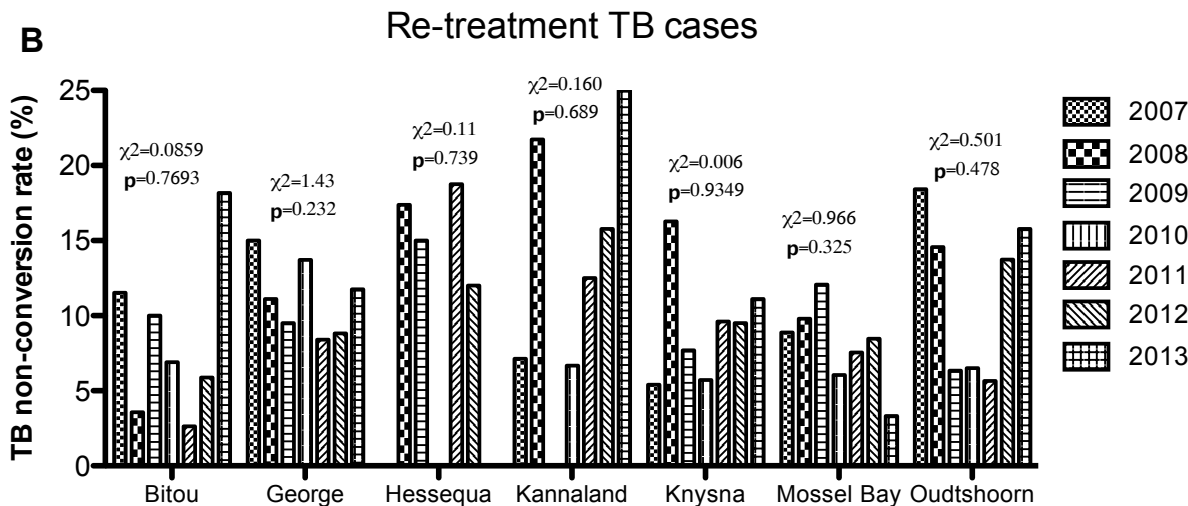

Supplement: Additional file 1: Figure S1. — Trends in tuberculosis sputum smear non-conversion rate in Eden sub-districts, Western Cape Province, 2007–2013. Chi-square trend for changes in the percentage of TB non-conversion and their respective p values are shown above in the graphs. (PDF 114 kb) [file 12879_2016_1712_MOESM1_ESM.pdf]

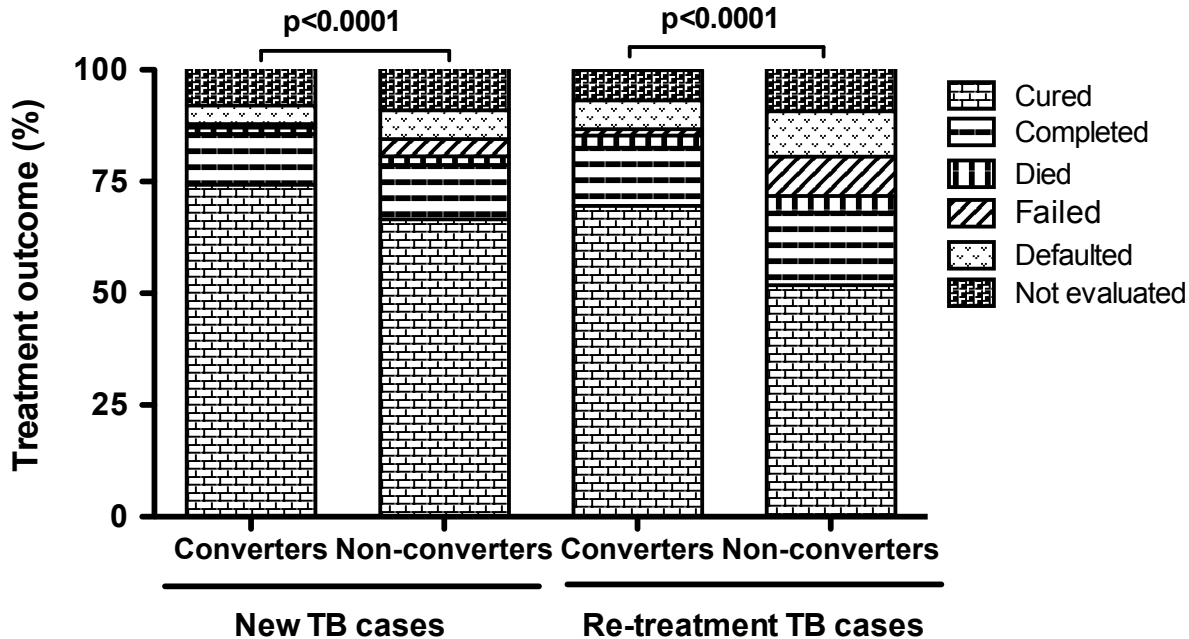

Supplement: Additional file 2: Figure S2. — Comparisons of treatment outcome in tuberculosis non-converters and converters of new and re-treatment TB cases. (PDF 49 kb) [file 12879_2016_1712_MOESM2_ESM.pdf]
